# Classifying the Information Needs of Survivors of Domestic Violence in Online Health Communities Using Large Language Models: Prediction Model Development and Evaluation Study

**DOI:** 10.2196/65397

**Published:** 2025-05-12

**Authors:** Shaowei Guan, Vivian Hui, Gregor Stiglic, Rose Eva Constantino, Young Ji Lee, Arkers Kwan Ching Wong

**Affiliations:** 1 Department of Electrical and Electronic Engineering The Hong Kong Polytechnic University Hung Hom China (Hong Kong); 2 Centre for Smart Health School of Nursing The Hong Kong Polytechnic University Hung Hom China (Hong Kong); 3 Department of Health and Community Systems School of Nursing University of Pittsburgh Pittsburgh, PA United States; 4 Faculty of Health Sciences University of Maribor Maribor Slovenia; 5 School of Nursing The Hong Kong Polytechnic University Hung Hom China (Hong Kong)

**Keywords:** domestic violence, help seeking, information needs, online health communities, large language models, generative artificial intelligence, multiclass text classification, artificial intelligence

## Abstract

**Background:**

Domestic violence (DV) is a significant public health concern affecting the physical and mental well-being of numerous women, imposing a substantial health care burden. However, women facing DV often encounter barriers to seeking in-person help due to stigma, shame, and embarrassment. As a result, many survivors of DV turn to online health communities as a safe and anonymous space to share their experiences and seek support. Understanding the information needs of survivors of DV in online health communities through multiclass classification is crucial for providing timely and appropriate support.

**Objective:**

The objective was to develop a fine-tuned large language model (LLM) that can provide fast and accurate predictions of the information needs of survivors of DV from their online posts, enabling health care professionals to offer timely and personalized assistance.

**Methods:**

We collected 294 posts from Reddit subcommunities focused on DV shared by women aged ≥18 years who self-identified as experiencing intimate partner violence. We identified 8 types of information needs: *shelters/DV centers/agencies*; *legal*; *childbearing*; *police*; *DV report procedure/documentation*; *safety planning*; *DV knowledge*; and *communication*. Data augmentation was applied using GPT-3.5 to expand our dataset to 2216 samples by generating 1922 additional posts that imitated the existing data. We adopted a progressive training strategy to fine-tune GPT-3.5 for multiclass text classification using 2032 posts. We trained the model on 1 class at a time, monitoring performance closely. When suboptimal results were observed, we generated additional samples of the misclassified ones to give them more attention. We reserved 184 posts for internal testing and 74 for external validation. Model performance was evaluated using accuracy, recall, precision, and *F*_1_-score, along with CIs for each metric.

**Results:**

Using 40 real posts and 144 artificial intelligence–generated posts as the test dataset, our model achieved an *F*_1_-score of 70.49% (95% CI 60.63%-80.35%) for real posts, outperforming the original GPT-3.5 and GPT-4, fine-tuned Llama 2-7B and Llama 3-8B, and long short-term memory. On artificial intelligence–generated posts, our model attained an *F*_1_-score of 84.58% (95% CI 80.38%-88.78%), surpassing all baselines. When tested on an external validation dataset (n=74), the model achieved an *F*_1_-score of 59.67% (95% CI 51.86%-67.49%), outperforming other models. Statistical analysis revealed that our model significantly outperformed the others in *F*_1_-score (*P*=.047 for real posts; *P*<.001 for external validation posts). Furthermore, our model was faster, taking 19.108 seconds for predictions versus 1150 seconds for manual assessment.

**Conclusions:**

Our fine-tuned LLM can accurately and efficiently extract and identify DV-related information needs through multiclass classification from online posts. In addition, we used LLM-based data augmentation techniques to overcome the limitations of a relatively small and imbalanced dataset. By generating timely and accurate predictions, we can empower health care professionals to provide rapid and suitable assistance to survivors of DV.

## Introduction

### Background

Domestic violence (DV) stands as a significant public health concern impacting many women through traumatic experiences and mental health conditions [1]. The World Health Organization estimates that 641 million women who have ever been married or partnered have reported experiencing physical or sexual intimate partner violence at least once since the age of 15 years [2]. The impact of DV goes beyond health problems and affects the economy as well [3]. According to the latest statistics from the Emergency Assistance Foundation in 2023 [4], DV results in approximately 8 million lost days of paid work annually, equivalent to >32,000 full-time jobs. Moreover, 96% of employed survivors of DV experience workplace difficulties due to the abuse, which may disrupt their productivity.

Traditional interventions for survivors of DV have focused on providing information, support, and counseling through in-person services such as shelters, support groups, and one-on-one therapy sessions [5]. While these interventions have been crucial in supporting survivors of DV, they often face limitations in reach and accessibility. Women facing DV frequently encounter in-person help-seeking barriers such as stigma, shame, guilt, and embarrassment, highlighting the challenges in accessing traditional in-person interventions [6]. In addition, factors such as geographical distance, lack of transportation, or fear of being discovered by abusers can further impede access to these services [7]. Moreover, traditional in-person interventions can be time-consuming and labor intensive, requiring significant resources to implement and maintain. This may result in a lack of personalized care [5]. However, providing personalized care to survivors of DV is crucial because each survivor faces unique circumstances and challenges [8,9], necessitating innovative approaches to meet the individual needs of survivors of DV effectively.

Online health communities (OHCs) have emerged as platforms where women experiencing DV can seek assistance and share their experiences. These OHCs offer unique advantages that address many of the limitations of traditional interventions. One of the most significant benefits is the anonymity that OHCs provide, allowing survivors to share their stories without fear of identification, which helps mitigate the stigma and shame often associated with DV. In addition, OHCs are accessible 24/7, enabling survivors to seek help and support at any time, unlike traditional services with restricted operating hours. The sense of community and peer support fostered in these online spaces is also therapeutic as it connects survivors with others who understand their experiences. The shared stories and needs within OHCs can be personalized across different groups of survivors of DV. A thorough understanding of their experiences is essential to ensure that the support provided is both effective and appropriate.

Moreover, the influence of social media advocacy movements such as X (formerly known as Twitter) hashtags such as #MeToo has amplified the voices of survivors of DV and facilitated a space for sharing experiences and seeking support [10,11]. Survivors often share their stories and express their help-seeking needs within their stories rather than asking for help directly. These OHC platforms, including X, provide real-time insights into the needs and experiences of survivors of DV, thereby overcoming the limitations of traditional methods and enabling the development of more responsive, tailored, and effective support services.

### Prior Work

To effectively understand the needs of survivors of DV and provide personalized support within OHCs, it is important to predict their information needs addressed within their online posts accurately. Current approaches for this task and other related topics in DV have leveraged machine learning techniques, such as the automatic identification of survivors of DV in critical need using deep learning and the identification of multiple classes of terms with the most common words from online posts using deep learning [12-14]. However, these methods often exhibit limited human language understanding capabilities and require substantial training datasets to achieve accurate predictions [15]. For multiclass text classification problems, one of the common deep learning methods is long short-term memory (LSTM), a type of recurrent neural network designed to effectively capture and retain information across long sequences. It is mainly used for tasks involving time-series data or sequential dependencies [16]. However, such methods require a substantial amount of well-labeled data for training, which are challenging to obtain for DV information need prediction. This challenge stems from the fact that manually analyzing and labeling the data is a time-consuming and labor-intensive task that requires qualified domain experts.

The rise of ChatGPT has garnered attention in providing tailored support through large language models (LLMs), which are a subset of machine learning approaches. LLMs have demonstrated the ability to understand nuances in human language and can be fine-tuned using human-designed prompts to provide tailored responses [17]. A key advantage of LLMs over traditional machine learning methods is their reduced requirement for extensive training data [17,18]. Unlike traditional approaches, which often necessitate large datasets for effective learning, LLMs benefit from extensive pretraining on vast amounts of data [15]. This pretraining enables them to develop a broad understanding of language, allowing them to be fine-tuned efficiently using smaller, domain-specific datasets [18]. Consequently, LLMs are particularly well suited for tasks for which large, well-labeled datasets are scarce, making them especially advantageous in complex domains such as DV intervention. Several studies have demonstrated the potential of LLMs in health care and mental health support. For instance, Lan et al [19] proposed an LLM-based method called Diagnostic Criteria–Guided Mood History Aware to detect depression from social media posts. Their method achieved high accuracy and explainability in identifying individuals who may be experiencing depression, outperforming other machine learning approaches. In addition, extracting data on social determinants of health from electronic health records via LLMs is more effective in providing better insights to support patients in need compared to traditional approaches [20]. While LLMs are highly used in text classification tasks across various domains, their application in health care, particularly in the DV domain, remains underexplored [21]. A knowledge gap still exists regarding the effectiveness of LLMs in performing multiclass text classification in the domain-specific area of DV.

One of the most frequent applications of LLMs is fine-tuning, a process that involves adapting a pretrained model using domain-specific data to enhance its performance on specialized tasks [22]. This approach enables LLMs to build on their broad, generalized knowledge base and refine their capabilities by focusing on the specific nuances of the new dataset, making them more effective for targeted applications [22]. In the health care sector, fine-tuning has been successfully used to customize LLMs for various tasks. For instance, Guevara et al [20] used fine-tuning to adapt an LLM to electronic health records, which consist of unstructured data in the form of free text, for the purpose of extracting data on social determinants of health, resulting in a model that was better aligned with the specific requirements of this task. In addition, the open-source ChatDoctor, which is based on the Llama model, was fine-tuned using 100,000 patient-physician conversations, leading to substantial improvements in the model’s ability to understand patient needs and provide accurate advice [23]. These examples demonstrate that fine-tuning LLMs using domain-specific data can significantly enhance their effectiveness in specialized domains, making them a valuable tool for addressing challenges in underresearched areas such as DV intervention.

An emerging application of LLMs is data augmentation, which enables these models to learn from existing data to generate new data, thereby addressing the challenges of limited data availability. This technique has gained widespread adoption in the field. For instance, Yuan et al [24] demonstrated that using a self-reward mechanism allows LLMs to augment data effectively, leading to improved performance in supervised fine-tuning. In addition, Chen et al [25] introduced a method called “self-play fine-tuning,” which empowers LLMs to generate their own training data based on the outcomes of previous iterations. Furthermore, directly instructing LLMs to mimic existing data to produce similar content has been successfully applied in generating health care and social media–related texts [26]. These examples demonstrate that using LLMs for data augmentation is an effective strategy for enhancing model performance and addressing data scarcity in various applications.

### Proposed Method

To address the gap in the application of LLMs for multiclass text classification in the domain-specific area of DV intervention, we proposed the adoption of an LLM-based approach in this study. Our methodology involved (1) fine-tuning the ChatGPT (GPT-3.5; OpenAI), Llama 2-7B (Meta Platforms), and Llama 3-8B (Meta Platforms) models specifically for the multiclass text classification within the DV domain; and (2) leveraging data augmentation techniques to enhance the prediction accuracy of the model. The dataset used was derived from OHCs related to DV, providing a rich source of information on survivors’ information needs.

### Goal and Research Questions

The overarching goal of this study was to fine-tune an LLM to provide fast and accurate predictions of information needed based on online posts of survivors of DV. The two research questions underpinning this goal are as follows: (1) can LLMs provide timely and accurate predictions of information needs based on online posts in the DV domain? (2) Which LLM (GPT-3.5, Llama 2-7B, or Llama 3-8B) is more effective in fine-tuning for multiclass text classification tasks, particularly in the context of DV information need support?

## Methods

### Overview

**Figure 1:**
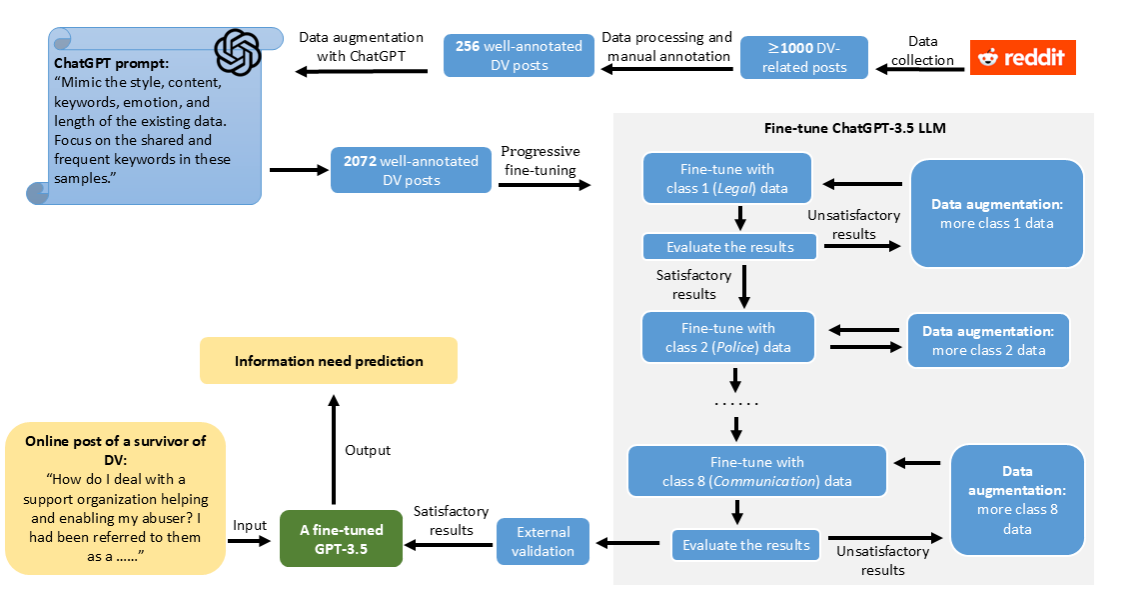
Workflow of the method development in this study. DV: domestic violence; LLM: large language model.

We collected data from specific subcommunities in Reddit and used GPT-3.5 to augment the dataset. A progressive training strategy was used to fine-tune the model, which was then evaluated on both real and artificial intelligence (AI)–generated posts. To further assess the generalization ability of our model, we conducted external validation on an independent dataset. The detailed methodology is outlined in the flowchart shown in Figure 1.

### Dataset

**Figure 2:**
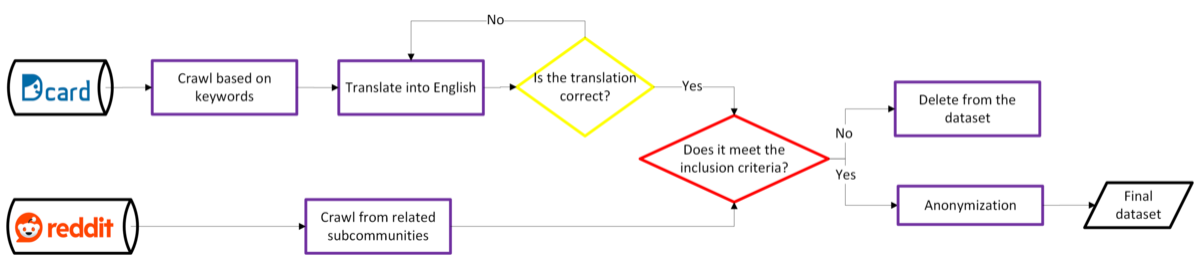
Workflow for the data collection and preprocessing of the 2 datasets.

The data used in this study were divided into 2 parts. The first part comprised the data used for model training and evaluation, whereas the second part consisted of an independent dataset that was separate from the training data, intended for external validation. Both datasets encompassed 8 distinct types of information needs: *shelters/DV centers/agencies*; *legal*; *childbearing*; *police*; *DV report procedure/documentation*; *safety planning*; *DV knowledge*; and *communication*. Figure 2 provides an overview of the workflow for the data collection and preprocessing of the 2 datasets.

The training and evaluation data were collected from a social networking site, Reddit, which is a platform for knowledge and information exchange based on a wide range of topics, including health-related ones, categorized into subreddit communities denoted using r/. This study extracted data from the subcommunities r/domesticviolence, r/abusiverelationship, and r/survivorsofabuse as the data source to understand how women asked for the information needed when they shared their intimate partner violence experience online [27]. Data collection involved crawling all posts within these subcommunities followed by the application of inclusion criteria to select only posts shared by women aged ≥18 years who self-identified as experiencing DV and sought advice. In the case of posts that identified a partner using pronouns such as “he/him/his” and described experiences without mentioning an age of <18 years, we regarded them as being from an adult woman. Conversely, posts written in non-English languages, those from underage women or women without abuse experience, posts lacking help-seeking attempts, and advertisements were excluded. All personal information such as user ID, names, and post location was not retrieved to adhere to the General Data Protection Regulation.

The external validation dataset was collected from a DV OHC on a social networking site, Dcard, in traditional Chinese. Data were extracted by searching for keywords such as “domestic violence,” “family violence,” and “domestic abuse” in post titles or main content. First, the posts were translated into English by the researchers with fluent bilingual ability, and then we applied the same inclusion criteria as for the training and evaluation data. To ensure trustworthiness, the translations were checked against the content back and forth multiple times. Meetings were held to reach an agreement with a third reviewer to ensure the credibility of the translations. The validation results were evaluated among team members before further fine-tuning to ensure credibility.

### Data Augmentation

Using GPT-3.5, we prompted the model to imitate the existing data within each class, focusing on replicating both the writing style and content to generate additional posts (refer to Textbox 1 for the prompt used). This approach ensured that the augmented data maintained the thematic relevance and authenticity of the original posts while increasing the overall size of the dataset. Our augmentation efforts resulted in a substantial expansion of the dataset, growing from 294 posts to 2216 posts, thereby providing a more comprehensive and representative corpus for model training and evaluation. In addition, the augmented data were cross-checked to ensure their relevance and authenticity, further validating the quality and applicability of the expanded dataset.

**The prompt used for data augmentation with GPT-3.5.**

I have {24} samples of posts from domestic violence survivors seeking {Legal} this kind of information support from online. Now, I want to generate {100} more samples that mimic the style, content, keywords, emotion, and length of the existing data also seeking {Legal} this kind of information support. You should focus on the shared and frequent keywords in these samples.

### Validation of AI-Generated Data

Given the critical importance of ensuring the relevance and authenticity of the AI-generated data, we implemented a 2-step validation process to evaluate the augmented dataset.

#### Step 1: Sampling and Manual Analysis

To begin, we performed a manual analysis on 10.2% (196/1922) of the sample of augmented data. This sampling was carefully stratified to cover each class of information needs identified in the study, with each class comprising approximately 10% of the total sample selected. In total, 2 domain experts independently reviewed the sampled data, focusing on the relevance, accuracy, and contextual integrity of the content. To ensure consistency and reliability in their assessments, we calculated the interrater reliability using the κ statistic. This metric provided a quantitative measure of agreement between the reviewers, highlighting the robustness of our validation process [28].

#### Step 2: Keyword Analysis

In addition, we performed a keyword analysis to ensure that the AI-generated posts contained the necessary key terms relevant to DV. This step was crucial in verifying that the augmented data preserved the essential thematic elements of the original posts, thereby maintaining the integrity of the dataset.

#### AI-Generated Data Quality

By using these 2 validation processes, we ensured that the augmented data were both high quality and fit for the purposes of our study. This approach not only enhanced the robustness of our findings but also provided a methodological framework that can be applied in future research involving AI-generated data.

### Fine-Tuning the GPT-3.5 LLM

We implemented a progressive training strategy to fine-tune the ChatGPT (GPT-3.5-turbo-0125) model using our dataset for this multiclass text classification. This involved training the model in one class at a time and sequentially moving to the next. This approach allowed us to closely monitor the model’s performance and identify any challenges or inconsistencies encountered during the training process. Throughout the training iterations, if suboptimal performance was observed between 2 classes, we applied data augmentation techniques to generate more samples that were similar to those misclassified samples. Thus, misclassified samples would be given more attention during the training process, whereas correctly classified ones would receive less attention.

We prompted the model using the prompt shown in Textbox 2 and iteratively fine-tuned the GPT-3.5 model using this progressive training strategy and incorporating data augmentation techniques. We aimed to optimize its performance for multiclass text classification, ensuring robustness and generalization across all classes in our dataset, thereby enhancing the model’s ability to accurately classify text samples into 1 of the 8 defined classes.

**The prompt used for the fine-tuning of GPT-3.5.**

You are a domestic violence analysis robot. Extract the information about domestic violence from the online posts below. We have 8 kinds of information needs. The following are the types and their definition: (A) Shelters/DV center/Agency: Any organizations working for domestic violence survivors, including shelters, community centers, agencies, NGOs, etc. (B) Legal: Information related to the legal system, laws, justice, and divorce procedure. (C) Childbearing: Information related to the rights to take care of children, and difficulties to take care of children. (D) Police: Information related to calling police for action, police reactions, police helpfulness. (E) DV report procedure/Documentation: Information related to how to report DV cases, what documents are needed, and what evidence is mandatory. (F) Safety planning: Information related to safety measures to protect the survivors, steps to leave the relationship safely, and plans for safety reporting. (G) DV knowledge: Seeking information about DV criteria, red flags, the cycle of DV, types of DV, etc. (H) Communication: Information related to the communication strategies with family, friends, children, and others during and after DV. Learning from the definition and return the class of the post from the above eight types.

### Data Analysis

#### Data Annotation

Drawing from the latest help-seeking literature by Sivagurunathan et al [29], a codebook was created by experts in the DV field to guide the annotation process. Subsequently, 2 undergraduate nursing student researchers annotated the dataset, with their work cross-checked by the principal investigator of this project to ensure quality. Any posts with ambiguous or unclear content underwent further discussion. Upon reaching saturation of codes for help-seeking behavior within the dataset, the domain experts finalized the codebook. This comprehensive annotation codebook included types of help received (such as informational and emotional support), networking opportunities offered by OHC members in their responses, and instances of experience sharing [27]. For this study, we focused on informational support needs identified in the original post and cross-checked the accuracy with the annotation codebook.

**Figure 3:**
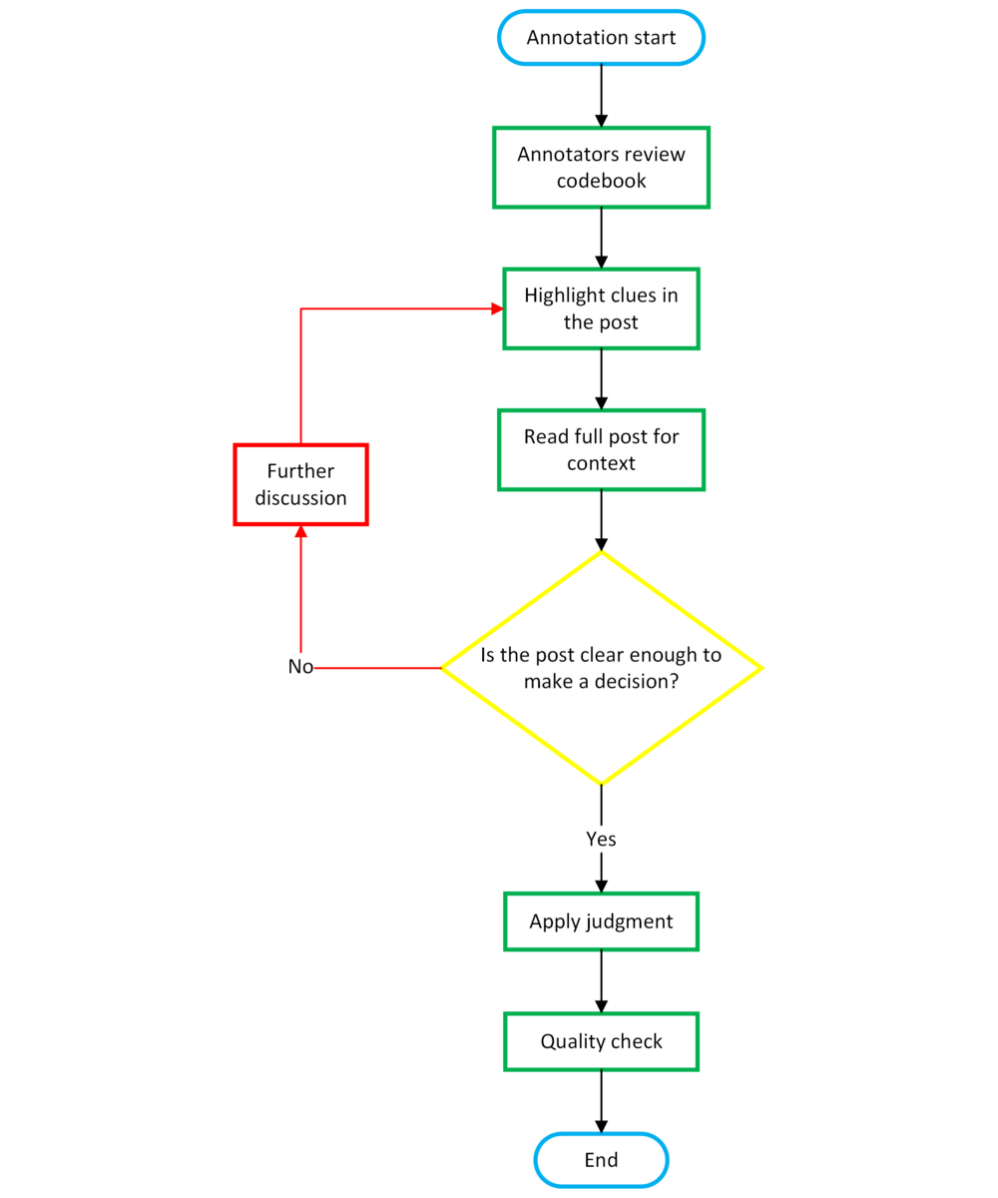
Workflow for the data annotation.

The specific steps of the annotation process were carefully designed to ensure accuracy and consistency in categorizing information needs, as shown in Figure 3. Initially, annotators reviewed the codebook, which provided detailed guidelines, definitions, and examples of typical keywords associated with each type of information need. This familiarization step was crucial for establishing a shared understanding of the annotation criteria.

Subsequently, annotators examined each individual post, beginning by highlighting potential clues based on the keywords outlined in the codebook. These keywords served as preliminary indicators for categorization (eg, terms such as “law” for *legal* needs or “shelter” for *shelters/DV centers/agencies* needs). The annotators then proceeded to read the entire post to evaluate its context, ensuring that their initial highlights aligned with the overall meaning and intent of the content.

If the context provided sufficient clarity, annotators applied their judgment, guided by the definitions and examples in the codebook, to assign the appropriate category of information need. However, if the content remained ambiguous or unclear, it was flagged for further discussion with a third researcher to reach a consensus. For quality assurance, all annotations were reviewed and cross-checked by the principal investigator, ensuring that the annotations met the required standards of accuracy and consistency.

#### LLM Evaluation

We applied our model to the test dataset to generate predictions and compare them with the ground-truth labels, which are the correct answers used to check the model’s accuracy. The test dataset consisted of 2 subsets: real posts and AI-generated posts. Real posts were used to evaluate how our model performed on actual, unseen cases in real-world scenarios, providing insights into its applicability and reliability when deployed in practice. These posts reflected diverse language use, writing styles, and genuine help-seeking behaviors of survivors of DV, offering a robust test for the model’s ability to handle varied inputs. Evaluating real posts is crucial for assessing the model’s ability to generalize from the training data to new, authentic examples. On the other hand, the AI-generated posts shared common features with the training dataset, allowing us to test the model’s performance in a controlled environment. This helps in identifying how well the model can handle content similar to what it has been trained on and ensures that it can maintain high performance on familiar patterns. In addition, AI-generated posts augment the diversity of the dataset by simulating various potential scenarios that might not be well represented in the limited real posts, thus enhancing the robustness of the model. Including both real and AI-generated posts ensures a comprehensive evaluation, covering both genuine, diverse user inputs and controlled, familiar patterns from the training set. This dual approach helps in thoroughly assessing the model’s performance, robustness, and generalization capabilities.

We set aside 13.6% (40/294) of real posts and 7.5% (144/1922) of AI-generated posts as a test set. The decision to allocate these specific percentages was guided by the inherent imbalance within our dataset. Given the limited number of real posts, we aimed to maximize the model’s exposure to these authentic examples to enhance its performance on real-world data. At the same time, we retained a portion of the real posts for testing to evaluate the model’s effectiveness in practical scenarios. The size of the test set was determined by the smallest subsets within the dataset, which is a common approach in imbalanced data settings to ensure that all classes are sufficiently represented in the evaluation phase [30]. Specifically, some subsets contained only 7 or 8 posts, so we selected 4 to 5 posts from each to ensure a more comprehensive evaluation. The remaining posts were included in the training dataset, allowing the model to better capture essential features of DV posts [30,31]. A similar principle was applied to the AI-generated posts, where we aimed to select approximately 20 posts from each subset, with the exact number varying slightly depending on the size of each subset. This approach allowed us to maintain a balanced and representative test set that could effectively assess the model’s performance across different categories.

We used various performance metrics, including accuracy, recall, precision, and *F*_1_-score, to assess our model’s performance. Accuracy is the proportion of correct predictions (both true positives and true negatives) out of all predictions.



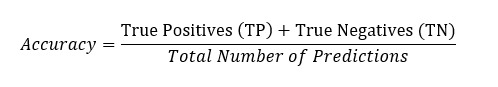



Recall, also known as sensitivity or true-positive rate, is the proportion of true instances of a class that are correctly identified by the model.



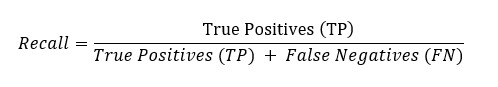



In our study, each class had only 2 possible outcomes: correct predictions or incorrect predictions. As a result, the number of true negatives for each class was 0, and the total number of predictions was equal to the sum of true positives and false negatives. Consequently, for each class, the accuracy metric was equivalent to the recall.



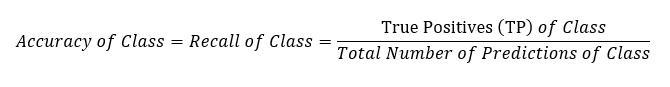



Precision, also known as positive predictive value, is the proportion of correct positive predictions out of all positive predictions made by the model.



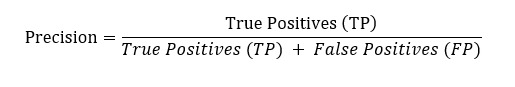



The *F*_1_-score is a crucial metric that integrates both precision and recall, accounting for the trade-off between them. It strikes a balance between these 2 metrics, making it particularly valuable in scenarios in which the dataset is imbalanced or when it is important to consider both precision and recall [32].



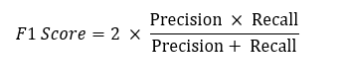



To evaluate the overall performance of the multiclass text classification model, we used macroaveraging, which involves calculating the average of the metrics for each class.



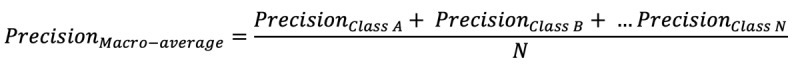





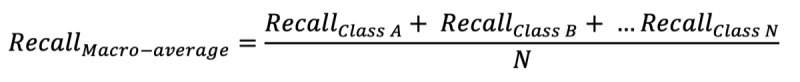





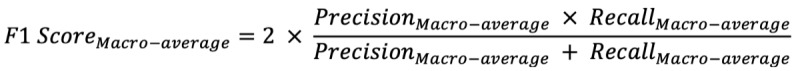



In this multiclass text classification task, accuracy can be used to evaluate the model’s overall prediction performance. However, accuracy alone may not fully reflect the model’s ability to distinguish between different classes, especially if the model exhibits biased behavior, such as consistently predicting the same class for all samples. In such cases, while accuracy may be high, precision for specific classes may be low as the model fails to make correct predictions for the other classes. A low precision indicates that the model is either overpredicting certain classes or unable to differentiate between them, suggesting potential model bias or class imbalance. Therefore, by examining the precision for each class, we can better assess whether the model is making biased predictions. For this reason, we chose *F*_1_-score as the final metric for comparison as it provides a more balanced evaluation by considering both precision and accuracy.

Our evaluation of the model was divided into 3 distinct comparisons to facilitate a comprehensive analysis. First, we compared the model with the original GPT-3.5 and GPT-4 models to demonstrate the efficacy of fine-tuning. Second, we compared its performance with that of the fine-tuned Llama 2-7B and Llama 3-8B models (epochs=3; learning rate=5 × 10^−5^; batch size=2) [33], and our model showcased a superior performance. The prompts used for these models were the same as those used for our model. We identified misclassified samples and presented them to domain experts for manual review. Experts analyzed the misclassifications to understand why the model made the wrong prediction, noting any language ambiguities or contextual misunderstandings. Finally, we timed the manual analysis process to compare it with the LLM’s prediction time, assessing our model’s efficiency. Although manual analysis took more time, the accuracy was quite high. To ensure fairness during this comparison, the fine-tuned model would detect the whole test set, but the data for manual analysis would only include those that had been correctly predicted by the fine-tuned model. To generalize the experiment, we recorded the prediction time for 5 rounds and computed the average as the final runtime of the model.

#### LLM External Validation

To rigorously assess the generalization capability of our model and further investigate its predictive ability, we applied it to an external validation dataset. This approach was crucial for evaluating the model’s effectiveness in real-world applications as it enabled us to determine how well the model could adapt to data that it had not encountered during training.

Notably, our training data were derived from Reddit, mainly in English, whereas our external validation data came from Dcard in traditional Chinese. This cultural and linguistic divergence presented an additional layer of complexity in our validation process. The differences in language structure, colloquial expressions, and cultural context may significantly influence how individuals express their experiences and needs related to DV.

We used the same evaluation metrics as in our initial assessments to ensure consistency and comparability of results. By using these standardized metrics (accuracy, recall, precision, and *F*_1_-score), we aimed to provide a thorough analysis of the model’s performance, highlighting its strengths and identifying potential areas for improvement. This cross-cultural evaluation not only tested the robustness of our model but also contributed valuable insights into the adaptability of LLMs in diverse contexts, particularly in sensitive areas such as DV.

### Ethical Considerations

Given that the web-based data used in this study were publicly accessible, an exemption approval was obtained from the institutional review board at the University of Pittsburgh (MOD20030179-002) and the Hong Kong Polytechnic University (HSEARS20230111007).

## Results

### Overview

Our research used a dataset comprising 2216 posts, including 294 (13.3%) real posts and 1922 (86.7%) AI-generated posts, as shown in Table 1. This dataset was used for both training and evaluation purposes. To evaluate the performance of our model, we set aside 13.6% (40/294) of real posts and 7.5% (144/1922) of AI-generated posts as a test set. The remaining 2032 posts, including 254 (12.5%) real posts and 1778 (87.5%) additional LLM-augmented posts, were used for fine-tuning the model. To ensure the reliability of our AI-generated posts, Cohen κ agreement was calculated on 10% of the selected posts from the 8 categories. The Cohen κ agreement obtained was 0.74, which indicates a substantial agreement between 2 annotators. This confirmed that the labels assigned to the generated posts were consistent with our requirements. In addition, we used an independent real-world dataset comprising 74 posts, as shown in Table 2, for external validation purposes.

**Table 1:** Overview of the dataset used for model training and evaluation (N=2216).

Information need class	Real posts (n=294), n (%)	AI^a^-generated posts (n=1922), n (%)	Overall dataset, n (%)
Shelters/DV^b^ centers/agencies	8 (2.7)	116 (6)	124 (5.6)
Legal	24 (8.2)	344 (17.9)	368 (16.6)
Childbearing	8 (2.7)	225 (11.7)	233 (10.5)
Police	8 (2.7)	335 (17.4)	343 (15.5)
DV report procedure/documentation	7 (2.4)	505 (26.3)	512 (23.1)
Safety planning	12 (4.1)	197 (10.2)	209 (9.4)
DV knowledge	66 (22.4)	148 (7.7)	214 (9.7)
Communication	161 (54.8)	52 (2.7)	213 (9.6)

^a^AI: artificial intelligence.

^b^DV: domestic violence.

**Table 2:** Overview of the external validation dataset (N=74).

Information need class	Posts, n (%)
Shelters/DV^a^ centers/agencies	6 (8)
Legal	6 (8)
Childbearing	4 (5)
Police	18 (24)
DV report procedure/documentation	8 (11)
Safety planning	5 (7)
DV knowledge	4 (5)
Communication	23 (31)

^a^DV: domestic violence.

In our research, we addressed the 2 proposed research questions. For the first research question—“Can LLMs provide timely and accurate predictions of information needs based on online posts in the DV domain?”—the analysis demonstrated that our fine-tuned LLM accurately predicted information needs, achieving an *F*_1_-score of 70.49% (95% CI 60.63%-80.35%) on the real post test set and 84.58% (95% CI 80.38%-88.78%) on the AI-generated post test set. Furthermore, for the external validation dataset, the model achieved an *F*_1_-score of 59.67% (95% CI 51.86%-67.49%). These results indicate that LLMs are effective in interpreting the nuances present in the posts of survivors of DV to identify specific information needs. Furthermore, the LLM completed the prediction of 40 posts in 19.108 seconds, in stark contrast to the 1150 seconds required for manual analysis. This significant reduction in processing time underscores the model’s capability to provide timely predictions of information needs.

For the second research question—“Which LLM (GPT-3.5, Llama 2-7B, or Llama 3-8B) is more effective in fine-tuning for multiclass text classification tasks, particularly in the context of DV information need support?”—our comparative analysis revealed that the fine-tuned GPT-3.5 model outperformed both Llama 2-7B and Llama 3-8B, achieving an *F*_1_-score of 70.49% (95% CI 60.63%-80.35%) on the real post test set, 84.58% (95% CI 80.38%-88.78%) on the AI-generated post test set, and 59.67% (95% CI 51.86%-67.49%) on the external validation dataset. Moreover, statistical analysis indicated that the *P* values for comparisons between GPT-3.5 (*P*<.001) and both Llama 2-7B (*P*=.047) and Llama 3-8B (*P*=.002) were <.05 for the real post test set and <.001 for the external validation dataset. These findings suggest that the fine-tuned GPT-3.5 model is the most effective among the evaluated models for addressing multiclass text classification tasks related to DV information needs.

The comprehensive comparative analysis was structured into 5 parts.

### Comparison With Original GPT-3.5 and GPT-4

**Figure 4:**
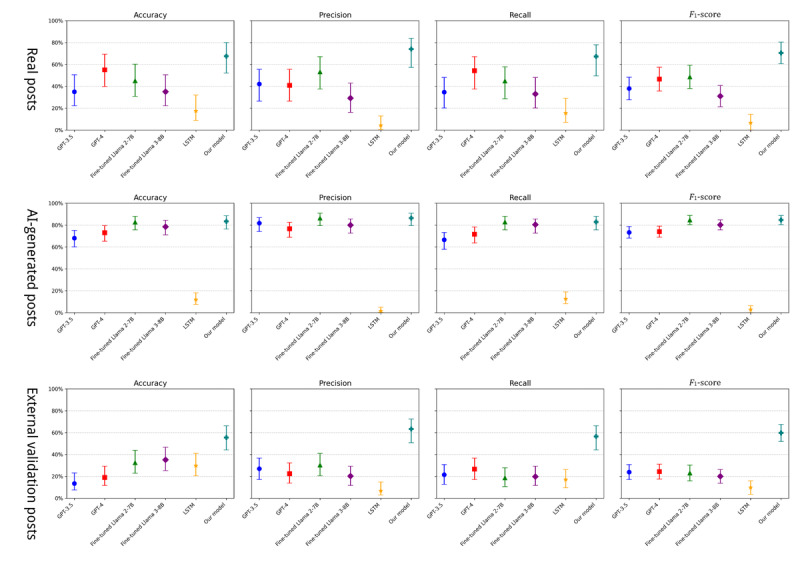
Model performance metrics across the real, artificial intelligence (AI)–generated, and external validation posts. LSTM: long short-term memory.

The first part of our analysis focused on comparing our fine-tuned model with the original GPT-3.5 and GPT-4 models. We used GPT-3.5 and GPT-4 directly using the application programming interface from OpenAI without any fine-tuning. For real posts, our fine-tuned model achieved a notable overall *F*_1_-score of 70.49% (95% CI 60.63%-80.35%), substantially outperforming the original GPT-3.5 (37.96%, 95% CI 27.69%-48.24%) and GPT-4 (46.54%, 95% CI 35.62%-57.45%) models, as shown in Table 3 and Figure 4. In addition, our model demonstrated superior performance in accuracy (67.5%), recall (67.29%), and precision (74.01%). With high precision, our model made more balanced predictions across 8 classes, avoiding biased predictions toward any particular class. Particularly, it exhibited substantial improvements in the prediction of childbearing, police, safety planning, DV knowledge, and communication information needs compared to the other models. In addition, there was a significant difference in model performance between the *F*_1_-scores of our model and of the original GPT-3.5 (*P*<.001) and between the *F*_1_-scores of our model and of the original GPT-4 (*P*=.03), as shown in Table 4, which suggests that both models demonstrated significantly lower performance than our fine-tuned model. For AI-generated posts, our model attained an *F*_1_-score of 84.58% (95% CI 80.38%-88.78%) compared to 73.33% (95% CI 68.03%-78.64%) for GPT-3.5 and 74.02% (95% CI 68.97%-79.07%) for GPT-4 (as shown in Table 5 and Figure 4). Similarly to the real posts, significant performance improvements were observed in the classes of childbearing, police, safety planning, and DV knowledge information needs. This demonstrates the effectiveness of the fine-tuning process as it substantially enhanced the model’s ability to predict the information needs of survivors of DV from both real and AI-generated online posts.

**Table 3:** Prediction results for information needs in the real post test set^a^.

Information need class			GPT-3.5		GPT-4		Fine-tuned Llama 2-7B		Fine-tuned Llama 3-8B		LSTM^b^		Our model
**Shelters/DV^c^ centers/agencies (n=5 test posts; %)**													
	Accuracy or recall	40		80		0		0		0		80	
	Precision	66.67		100		0		0		0		100	
	*F*_1_-score	50		88.89		0		0		0		88.89	
**Legal (n=5 test posts; %)**													
	Accuracy or recall	20		40		20		20		0		60	
	Precision	33.33		40		100		33.33		0		50	
	*F*_1_-score	25		40		33.33		25		0		54.55	
**Childbearing (n=4 test posts; %)**													
	Accuracy or recall	0		0		75		0		0		75	
	Precision	0		0		75		0		0		75	
	*F*_1_-score	0		0		75		0		0		75	
**Police (n=5 test posts; %)**													
	Accuracy or recall	20		40		60		40		0		60	
	Precision	100		33.33		75		40		0		75	
	*F*_1_-score	33.33		36.36		66.67		40		0		66.67	
**DV report procedure/documentation (n=5 test posts; %)**													
	Accuracy or recall	40		80		0		0		40		40	
	Precision	20		36.36		0		0		11.76		66.67	
	*F*_1_-score	26.67		50		0		0		18.18		50	
**Safety planning (n=5 test posts; %)**													
	Accuracy or recall	80		80		60		60		0		60	
	Precision	33.33		33.33		100		75		0		100	
	*F*_1_-score	47.06		47.06		75		66.67		0		75	
**DV knowledge (n=5 test posts; %)**													
	Accuracy or recall	60		80		60		60		0		80	
	Precision	33.33		50		50		60		0		80	
	*F*_1_-score	42.85		61.54		54.55		60		0		80	
**Communication (n=6 test posts; %)**													
	Accuracy or recall	16.67		33.33		83.33		83.33		83.33		83.33	
	Precision	50		33.33		25		25		21.74		45.45	
	*F*_1_-score	25		33.33		38.46		38.46		34.48		58.82	
**Total (n=40 test posts), point estimate (95% CI)**													
	Accuracy	35.00 (22.13-50.49)		55.00 (39.83-69.29)		45.00 (30.71-60.17)		35.00 (22.13-50.49)		17.50 (8.75-31.95)		67.50 (52.02-79.92)	
	Recall	34.58 (20.08-47.98)		54.17 (37.50-67.06)		44.79 (28.51-57.80)		32.92 (20.08-47.98)		15.42 (7.06-29.07)		67.29 (49.51-77.87)	
	Precision	42.08 (26.35-55.40)		40.79 (26.35-55.40)		53.13 (37.50-67.06)		29.17 (16.11-42.83)		4.19 (0.44-12.88)		74.01 (57.17-83.89)	
	*F*_1_-score	37.96 (27.69-48.24)		46.54 (35.62-57.45)		48.60 (37.99-59.22)		30.93 (21.22-40.65)		6.59 (0.00-14.35)		70.49 (60.63-80.35)	

^a^All values are rounded to 2 decimal places.

^b^LSTM: long short-term memory.

^c^DV: domestic violence.

**Table 4:** *P* values for model comparisons regarding F1-score across real, artificial intelligence (AI)–generated, and external validation posts.

	Real posts, *P* value	AI-generated posts, *P* value	External validation posts, *P* value
GPT-3.5	<.001	.24	<.001
GPT-4	.03	.38	<.001
Fine-tuned Llama 2-7B	.047	.97	<.001
Fine-tuned Llama 3-8B	.002	.91	<.001
LSTM^a^	<.001	<.001	<.001

^a^LSTM: long short-term memory.

**Table 5:** Prediction results for information needs in the artificial intelligence–generated post test set^a^.

Information need class			GPT-3.5		GPT-4		Fine-tuned Llama 2-7B		Fine-tuned Llama 3-8B		LSTM^b^		Our model
**Shelters/DV^c^ centers/agencies (n=20 test posts; %)**													
	Accuracy or recall	100		100		100		100		0		100	
	Precision	100		100		100		100		0		100	
	*F*_1_-score	100		100		100		100		0		100	
**Legal (n=16 test posts; %)**													
	Accuracy or recall	100		100		100		93.75		0		100	
	Precision	69.57		69.57		84.21		71.43		0		64	
	*F*_1_-score	82.05		82.05		91.43		81.08		0		78.05	
**Childbearing (n=16 test posts; %)**													
	Accuracy or recall	56.25		50		100		93.75		0		75	
	Precision	100		88.89		88.89		93.75		0		92.31	
	*F*_1_-score	72		64		94.12		93.75		0		82.76	
**Police (n=16 test posts; %)**													
	Accuracy or recall	18.75		56.25		100		100		0		100	
	Precision	100		100		100		94.12		0		100	
	*F*_1_-score	31.58		72		100		96.97		0		100	
**DV report procedure/documentation (n=17 test posts; %)**													
	Accuracy or recall	5.88		11.76		17.65		29.41		100		11.76	
	Precision	100		66.67		100		83.33		11.81		100	
	*F*_1_-score	11.11		19.99		30		43.48		21.12		21.05	
**Safety planning (n=13 test posts; %)**													
	Accuracy or recall	100		100		100		100		0		100	
	Precision	30.23		46.43		76.47		68.42		0		68.42	
	*F*_1_-score	46.43		63.42		86.67		81.25		0		81.25	
**DV knowledge (n=20 test posts; %)**													
	Accuracy or recall	55		55		45		65		0		80	
	Precision	64.71		68.75		75		61.9		0		80	
	*F*_1_-score	59.46		61.11		56.25		63.41		0		80	
**Communication (n=26 test posts; %)**													
	Accuracy or recall	96.15		100		100		61.54		0		96.15	
	Precision	89.29		72.22		66.67		66.67		0		86.21	
	*F*_1_-score	92.59		83.87		80		64		0		90.91	
**Total (n=144 test posts), point estimate (95% CI)**													
	Accuracy	68.06 (60.06-75.12)		72.92 (65.13-79.51)		82.64 (75.63-87.96)		78.47 (71.07-84.40)		11.81 (7.50-18.09)		83.33 (76.40-88.54)	
	Recall	66.50 (57.91-73.21)		71.63 (63.67-78.26)		82.83 (75.63-87.96)		80.43 (72.57-85.60)		12.50 (8.06-18.89)		82.86 (75.63-87.96)	
	Precision	81.73 (74.09-86.78)		76.57 (68.82-82.58)		86.40 (79.52-90.83)		79.95 (72.57-85.60)		1.48 (0.38-4.92)		86.37 (79.52-90.83)	
	*F*_1_-score	73.33 (68.03-78.64)		74.02 (68.97-79.07)		84.58 (80.37-88.78)		80.19 (75.59-84.79)		2.65 (0.00-6.28)		84.58 (80.38-88.78)	

^a^All values are rounded to 2 decimal places.

^b^LSTM: long short-term memory.

^c^DV: domestic violence.

### Comparison With the Fine-Tuned Llama 2-7B and Llama 3-8B Models

The second part of our analysis compared the performance of our fine-tuned GPT-3.5 model with fine-tuned versions of the Llama 2-7B and Llama 3-8B models. The data used to fine-tune both Llama 2-7B and Llama 3-8B consisted of the same dataset of 2032 posts used for our model. However, unlike our approach, the fine-tuning of these 2 models did not incorporate a progressive training strategy. This difference in methodology may have implications for the performance of the models in addressing multiclass text classification tasks related to DV information needs. For real posts, our fine-tuned model demonstrated a marked performance improvement, with an overall *F*_1_-score of 70.49% (95% CI 60.63%-80.35%) on the test set. In comparison, the fine-tuned Llama 2-7B and Llama 3-8B models achieved *F*_1_-scores of 48.6% (95% CI 37.99%-59.22%) and 30.93% (95% CI 21.22%-40.65%), respectively, as shown in Table 3 and Figure 4. Notably, our model excelled in predicting the *shelters/DV centers/agencies* and *DV report procedure/documentation* classes, in which both Llama 2-7B and Llama 3-8B models failed to make any correct predictions (*F*_1_-score=0%). However, our model achieved *F*_1_-scores of 88.89% for the *shelters/DV centers/agencies* class and 50% for *DV report procedure/documentation* class. In addition, improvements were observed in the *legal*, *DV knowledge*, and *communication* classes. Moreover, our model demonstrated a significantly higher performance than that of the fine-tuned Llama 2-7B and Llama 3-8B models. Specifically, the differences in *F*_1_-scores were statistically significant, with *P* values of .047 for the fine-tuned Llama 2-7B and .002 for the fine-tuned Llama 3-8B, as illustrated in Table 4. These findings suggest that both Llama models performed notably worse than our fine-tuned model in real posts. For AI-generated posts, our model achieved an *F*_1_-score of 84.58% (95% CI 80.38%-88.78%) compared to 84.58% (95% CI 80.37%-88.78%) for Llama 2-7B and 80.19% (95% CI 75.59%-84.79%) for Llama 3-8B, as shown in Table 5 and Figure 4. While our model had a similar performance to that of Llama 2-7B, it outperformed Llama 3-8B. Considering both real and AI-generated posts, our fine-tuned GPT-3.5 model outperformed the other fine-tuned models overall. This superior performance indicates that our fine-tuning approach and model architecture are more effective in capturing the nuances of the information needs of survivors of DV within OHCs.

### Comparison With the Traditional Deep Learning Method

**Figure 5:**
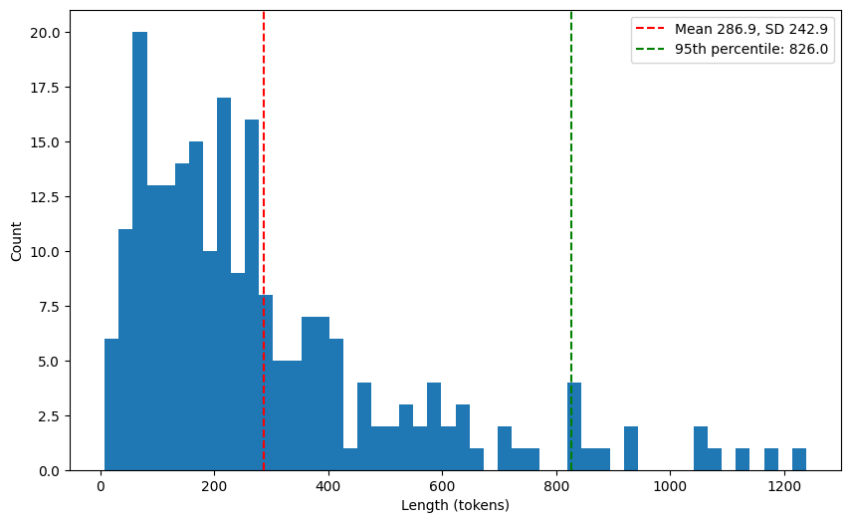
Distribution of text lengths in the real post dataset.

To investigate whether LLMs outperform traditional methods such as LSTM, we used the same training dataset of 2032 posts for LSTM training. LSTM is widely recognized for its effectiveness in text classification tasks, particularly in capturing relationships between words over long contexts [34]. In this study, we used a structure consisting of 2 LSTM layers followed by 3 dense layers. The maximum input length was set to 512, with a batch size of 64 and 30 epochs. This maximum length was determined based on the distribution of text lengths in our real post dataset to include most samples (as shown in Figure 5).

However, the LSTM model struggled to produce satisfactory predictions for both real and AI-generated posts, achieving *F*_1_-scores of just 6.59% (95% CI 0%-14.35%) and 2.65% (95% CI 0%-6.28%), respectively. There was a significant difference in model performance between the *F*_1_-scores of our model and of LSTM (*P*<.001), as shown in Table 4. It was only able to make predictions in the *DV report procedure/documentation* and *communication* classes, as shown in Tables 3 and 5, which are the dominant classes in the training dataset. Notably, the *DV report procedure/documentation* class contained the largest number of samples with 490 posts, whereas the *communication* class had the highest number of real posts available for training (155 posts).

These results highlight the limitations of traditional deep learning methods, particularly in multiclass text classification tasks influenced by imbalanced datasets. In addition, LSTMs impose strict requirements on input lengths, which may affect performance as padding or truncating text may alter its original meaning.

### Comparison Using the External Validation Dataset

To evaluate the generalization ability of our model, we conducted a comparison between all baseline models and our model using an independent dataset. Our model demonstrated the best performance, achieving an *F*_1_-score of 59.67% (95% CI 51.86%-67.49%), as shown in Table 6. There were significant differences in *F*_1_-scores compared to the other models: *P*<.001 for GPT-3.5, *P*<.001 for GPT-4, *P*<.001 for fine-tuned Llama 2-7B, *P*<.001 for fine-tuned Llama 3-8B, and *P*<.001 for LSTM. Importantly, our model successfully made predictions across all classes, whereas the other models failed to predict certain specific classes. This indicates our model’s robust predictive capabilities. Furthermore, given the characteristics of the external validation dataset, which was in traditional Chinese and reflected cultural contexts distinct from those of our training data, our model continued to demonstrate stable performance. This underlines its generalization ability to real-world scenarios, enhancing its relevance for practical applications. Overall, the comparison illustrates not only the technical superiority of our model over the baseline approaches but also its ability to generalize well to diverse, real-world scenarios, further validating its potential for deployment in practical applications.

**Table 6:** Prediction results for information needs in the external validation test set^a^.

Information need class			GPT-3.5		GPT-4		Fine-tuned Llama 2-7B		Fine-tuned Llama 3-8B		LSTM^b^		Our model
**Shelters/DV^c^ centers/agencies (n=6 test posts; %)**													
	Accuracy or recall	66.67		16.67		0		0		0		50	
	Precision	12.5		33.33		0		0		0		100	
	*F*_1_-score	21.05		22.22		0		0		0		66.67	
**Legal (n=6 test posts; %)**													
	Accuracy or recall	0		33.33		0		0		0		50	
	Precision	0		22.22		0		0		0		37.5	
	*F*_1_-score	0		26.67		0		0		0		42.86	
**Childbearing (n=4 test posts; %)**													
	Accuracy or recall	25		25		25		0		0		75	
	Precision	14.29		12.5		100		0		0		33.33	
	*F*_1_-score	18.18		16.67		40		0		0		46.15	
**Police (n=18 test posts; %)**													
	Accuracy or recall	5.56		27.78		16.67		16.67		0		55.56	
	Precision	50		83.33		100		100		0		83.33	
	*F*_1_-score	10		41.67		28.57		28.57		0		66.67	
**DV report procedure/documentation (n=8 test posts; %)**													
	Accuracy or recall	0		0		0		0		62.5		50	
	Precision	0		0		0		0		20		80	
	*F*_1_-score	0		0		0		0		30.3		61.54	
**Safety planning (n=5 test posts; %)**													
	Accuracy or recall	20		60		0		0		0		40	
	Precision	5.55		18.75		0		0		0		66.67	
	*F*_1_-score	8.7		28.57		0		0		0		50	
**DV knowledge (n=4 test posts; %)**													
	Accuracy or recall	50		50		25		50		0		75	
	Precision	33.33		9.09		11.11		28.57		0		60	
	*F*_1_-score	40		15.38		15.38		36.36		0		66.67	
**Communication (n=23 test posts; %)**													
	Accuracy or recall	4.35		0		82.61		91.3		73.91		56.52	
	Precision	100		0		31.15		33.33		34.69		44.83	
	*F*_1_-score	8.33		0		45.24		48.84		47.22		50	
**Total (n=74 test posts), point estimate (95% CI)**													
	Accuracy	13.51 (7.51-23.12)		18.92 (11.62-29.29)		32.43 (22.86-43.73)		35.14 (25.24-46.50)		29.73 (20.53-40.93)		55.41 (44.09-66.18)	
	Recall	21.45 (12.69-30.79)		26.60 (17.10-36.65)		18.66 (10.56-27.77)		19.75 (11.62-29.29)		17.05 (9.53-26.24)		56.51 (44.09-66.18)	
	Precision	26.96 (17.10-36.65)		22.40 (13.77-32.27)		30.28 (20.53-40.93)		20.24 (11.62-29.29)		6.84 (2.92-14.86)		63.21 (50.77-72.35)	
	*F*_1_-score	23.89 (17.09-30.69)		24.32 (17.51-31.13)		23.09 (15.87-30.31)		19.99 (13.74-26.24)		9.76 (3.53-16.00)		59.67 (51.86-67.49)	

^a^All values are rounded to 2 decimal places.

^b^LSTM: long short-term memory.

^c^DV: domestic violence.

### Speed Comparison With Manual Analysis

The average prediction time of our model was 19.108 seconds (SD 6.730; the values of 5 rounds of testing were as follows: 28.425, 23.736, 16.021, 15.172, and 12.185 seconds) for the entire real post test set of 40 posts, which significantly outperformed manual analysis. The manual analysis by experts (VH and RC) took approximately 1150 seconds to analyze 27 posts, which were the correctly predicted subsets of the real post test set. This highlighted the LLM’s efficiency in processing text data compared to manual methods, demonstrating its potential to expedite tasks requiring text classification.

## Discussion

### Principal Findings

Our dataset for model training and evaluation comprised 2216 posts, consisting of 294 (13.3%) real posts and 1922 (86.7%) AI-generated posts. This dataset is particularly significant as it captures a diverse range of experiences and needs expressed by survivors of DV within OHCs. It encompasses a broad spectrum of situations that may not be adequately represented in traditional data sources such as face-to-face interviews. Unlike typical demographic studies, which often focus on specific populations or geographic regions [35], our dataset integrates voices from individuals across various cities and regions, allowing for a more diverse representation of experiences. This diversity in our dataset showcases the varied nature of DV experiences and makes our findings more applicable to a wide range of real-world situations. Revisiting our research questions, we sought to determine whether LLMs could provide timely and accurate predictions of information needs and which model would be most effective in this task. Our study is the first to use a fine-tuned LLM for predicting the information needs of survivors of DV from OHCs. The value of OHCs lies in their ability to capture real-time experiences and needs [36], which our fine-tuned GPT-3.5 leveraged to significantly improve prediction accuracy and speed. We demonstrated a remarkable enhancement in performance, increasing accuracy from 35% (95% CI 22.13%-50.49%) to 67.5% (95% CI 52.02%-79.92%) in the real post test set, offering a much more efficient alternative to manual analysis.

Compared with traditional deep learning methods such as LSTM networks, which are highly used in natural language processing, LLMs significantly improve model prediction results in multiclass text classification tasks, particularly in addressing the long-tail problem. In our dataset, we observed a clear long-tail distribution in which certain DV post types appeared much less frequently (forming the “tail” classes) compared to dominant types (forming the “head” classes). LSTMs heavily rely on domain-specific training datasets, meaning that the model’s parameters are learned solely from the provided data. This can lead to biased predictions, especially when the dataset exhibits such a long-tail distribution in which tail classes have limited training examples [34]. Traditional models tend to perform well on head classes but fail to generalize to tail classes due to insufficient learning signals. In our study, which focused on DV posts on social media, survivors often shared personal stories and emotions without the use of specific keywords, making tail classes even harder to identify. This point is different from another application of LSTMs in sentiment analysis, which typically involves shorter texts that contain keywords and phrases [37]. As a result, traditional deep learning models, which learn strictly from the training data, may fail to capture these subtle emotional expressions and are prone to biases, especially toward the majority class in the dataset. In contrast, pretrained LLMs benefit from exposure to vast amounts of diverse data. For example, GPT-3.5 is trained on 499 billion tokens [17]. This pretraining allows LLMs to handle nuanced language and diverse contextual cues that traditional models struggle with, even with limited examples.

Furthermore, a key limitation of deep learning models, including LSTMs, is the restriction on token length. In our experiment, we set the maximum token length to 512 tokens. However, as illustrated in Figure 5, some posts exceeded this length, whereas others remained below it. When padding or truncating posts, important information may be lost or distorted, potentially introducing noise that negatively impacts both training and testing. On the other hand, LLMs do not face such limitations and can handle texts of varying lengths directly without any preprocessing [38], preserving all the relevant information. This makes LLMs not only more efficient in retaining the integrity of the original content but also better suited for real-world applications in which text length can vary widely. By overcoming these limitations, LLMs offer a more robust and convenient solution for long text classification tasks, such as DV posts, providing a significant improvement over traditional deep learning approaches.

Another notable finding of our study is that we could extract and identify information needs from DV posts with greater precision, yielding a total of 8 distinct types of DV information needs. This advancement is particularly significant as it enables a more tailored approach to support services, effectively addressing the specific requirements of survivors. Previous research has often encountered challenges in categorizing such needs due to the complexity and variability inherent in individuals’ expressions of their experiences [14]. The model’s improved accuracy in classifying these needs represents a substantial advancement in the field, yielding valuable insights that can inform the practice. This achievement can be attributed to the development of a well-annotated database of 294 DV posts, which encompassed all 8 types of information needs. Furthermore, the application of data augmentation technology enabled us to further enhance the dataset. The inherent limitations of our original dataset, characterized by its small size and imbalance, presented significant obstacles in training robust models. To overcome these challenges, we leveraged GPT-3.5 for data augmentation, generating additional posts to enrich the dataset and enhance its diversity. This approach was carefully selected to ensure that the augmented data retained the semantic integrity and nuanced context of posts related to DV. Previous methodologies, such as the easy data augmentation (EDA) techniques proposed by Wei and Zou [39] for text classification tasks, have demonstrated varying degrees of success in similar scenarios. EDA proves especially effective when applied to smaller datasets. Training with EDA using just half of the available training data achieved the same accuracy as standard training using the full dataset. In the health care domain, Yuan et al [40] successfully used LLMs for data augmentation to improve the matching of patients with clinical trials. Our study extends these methodologies by prioritizing semantic fidelity in the context of DV-related content, underscoring the critical importance of context preservation in data augmentation. Another possible contributing factor to the improved classification precision is the use of LLMs, which facilitate a focus on sentence meanings. In contrast to previous research, such as the deep learning approach used by Subramani et al [14] that classified DV posts into 5 broad and general categories, our study demonstrates a more in-depth and fine-grained understanding of DV information needs through the application of fine-tuned LLMs. While previous studies have focused on classifying DV posts into general categories, our approach enabled us to extract more specific and actionable information from the text, providing a more detailed and informative framework for understanding DV information needs. This advancement has significant implications for the development of more effective support systems for survivors of DV.

Our research also achieved a significant breakthrough by enabling the analysis of DV-related text without the need for structured data. This was made possible by the use of LLMs, which can directly process and extract valuable insights from unstructured text data. This capability is attributed to the LLMs’ ability to learn nuanced language patterns and relationships, allowing them to identify and categorize DV-related information with high accuracy. In contrast, previous studies have relied on structured data and numerical features extracted from DV arraignments to inform arraignment decisions [41]. This approach requires manual effort to extract and structure the relevant information, which can be time-consuming and labor intensive. Our approach, on the other hand, can analyze large volumes of unstructured text data with minimal human intervention, making it a more efficient and effective solution for extracting insights from DV-related text information.

In addition, our method has the potential to be applied to a wide range of tasks and domains. For example, the method used in this study can be applied to the task of study description harmonization, which involves standardizing and harmonizing study descriptions in medical imaging datasets [42]. By using a similar approach to the one used in this study, researchers can develop models that can accurately and efficiently harmonize study descriptions, enabling more effective cohort selection and data analysis. The generality of our method is also demonstrated by its ability to be fine-tuned for specific tasks and datasets. By using a progressive training strategy and generating additional samples to address misclassifications, the model can be adapted to a wide range of tasks and datasets, enabling its application to a variety of domains and use cases.

### Clinical Implications

This study illustrates the potential to enhance the assessment of information needs for women experiencing DV through the application of LLMs from a clinical perspective. With the increasing prevalence of electronic health records and patient portals, health care professionals can use the methodologies presented in our approach to provide timely and relevant resources to survivors via a recommendation system. From a practical standpoint, for instance, clinicians could implement a system that automatically identifies and prioritizes resources related to legal assistance, mental health support, and emergency services based on the specific needs expressed by survivors of DV. This will enable frontline health care professionals (ie, community nurses, social workers, or counselors) to offer prompt and personalized assistance through the health information system in clinical settings or community-based settings in nongovernmental organizations. Consequently, when survivors express any type of information need through patient portals or digital patient diaries via text, they could receive automated responses with pertinent and validated resources, thereby reducing their waiting time and enhancing their satisfaction with the personalized support provided.

In the context of DV-related nongovernmental organizations, our approach can be integrated into their peer support network forums or AI chatbot development. For example, an AI chatbot could be programmed to respond to common queries about safety resources, emotional support, and legal rights, ensuring that survivors receive accurate information promptly. Although the accuracy of our fine-tuned model currently stands at approximately 70%, the findings of this study demonstrate the feasibility of automatically prioritizing information needs using LLMs. Therefore, frontline social workers or counselors could rely on the automated system to address cases that cannot be easily classified or that are potentially life-threatening, thereby reducing the workload and stress associated with manual case review.

In terms of future health research in this area, DV agencies, health care providers, and survivors of DV could cocreate and validate the prediction from LLMs. For example, Hui et al [27] identified that both emotional and informational needs from OHCs were extractable. However, our study only focused on the accuracy of the information needed. To deliver a holistic care approach for survivors of DV, cocreation and validation with concerned parties regarding the LLM prediction results regarding both informational and emotional needs could greatly improve the practicality, applicability, feasibility, and usefulness of our proposed model.

### Limitations and Future Work

The first limitation is the diversity and generalizability of the dataset. As we only used posts from Reddit for model training, the applicability and generalizability of our proposed model to other datasets or platforms might be limited to English-speaking countries. This limitation is evident in our external validation, which involved content in traditional Chinese, where the model exhibited reduced accuracy. In addition, the demographic information from our dataset was relatively limited; we relied on the self-disclosure of the original posters to identify whether they were women aged ≥18 years. Some users may hide their actual sex and age information due to privacy issues in OHCs. Although LLM-based data augmentation was used to enrich the dataset, some of the generated posts closely resembled the training data with limited diversity. This lack of variability in AI-generated data may affect the model’s generalizability to real-world data as such synthetic data often lack the quality necessary for robust training.

The second limitation is the model’s inability to extract multiple types of information needs concurrently. A manual inspection of inaccurate predictions revealed that many so-called errors were partial predictions. This indicates that a single post frequently encompasses several types of information needs, but the model could only extract one type at a time, which occasionally diverged from the true label.

Future work should focus on collecting more diverse data from various OHCs and incorporating them into the training dataset. In addition, methods to enhance the LLM’s capability to identify and extract multiple types of information needs simultaneously should be explored.

### Conclusions

Our study used a progressive approach to fine-tune a multiclass text classification LLM using data augmentation on a relatively small and unbalanced dataset. This approach allowed us to extract and identify information needs related to DV from online posts effectively and swiftly, which offers a promising solution for empowering health care professionals to provide timely and personalized assistance to survivors of DV.

## Abbreviations

AI: artificial intelligence
DV: domestic violence
EDA: easy data augmentation
LLM: large language model
LSTM: long short-term memory
OHC: online health community
